# Supplementary material for: Molecular determinants of μ-conotoxin KIIIA interaction with the human voltage-gated sodium channel NaV1.7
Source: Front Pharmacol. 2023 Mar 16;14:1156855. doi: 10.3389/fphar.2023.1156855 (PMC10060530; doi:10.3389/fphar.2023.1156855)

Supplementary Material for:

Molecular Determinants of μ-Conotoxin KIIIA Interaction with the Human Voltage-Gated Sodium Channel $Na_V1.7$

Ian H. Kimball[1,*], Phuong T. Nguyen[1,*], Baldomero M. Olivera[3],

Jon T. Sack[1,2,‡], Vladimir Yarov-Yarovoy[1,2,‡]

[1]Department of Physiology and Membrane Biology, UC Davis, Davis, CA, USA,

[2]Department of Anesthesiology and Pain Medicine, UC Davis, Davis, CA, USA,

[3]Department of Biology, University of Utah, Salt Lake City, UT, USA

* - these authors contributed equally to this work

‡ corresponding authors

Supplementary Figure 1—Supplement 1

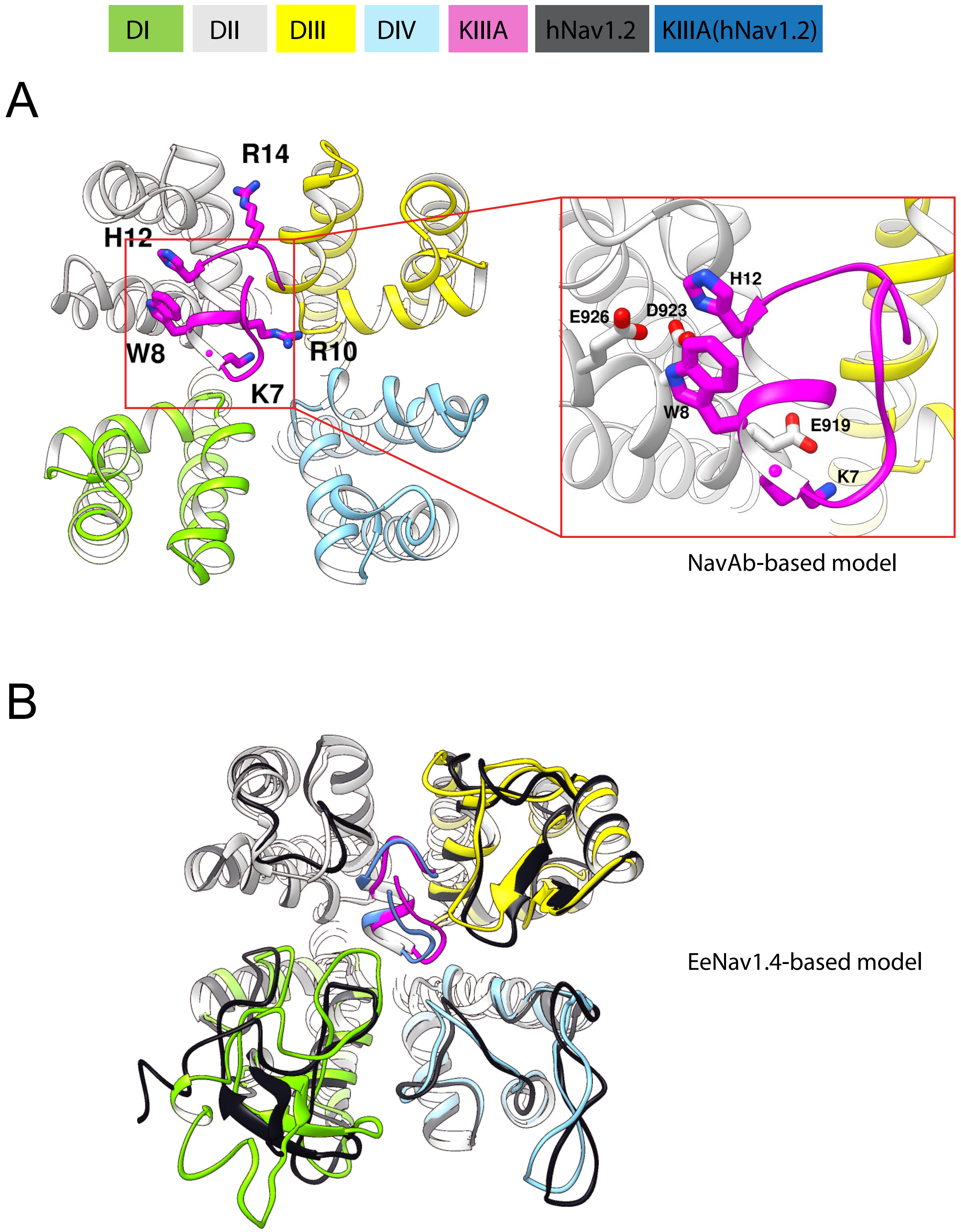

Supplementary Figure 2 —Supplement 1

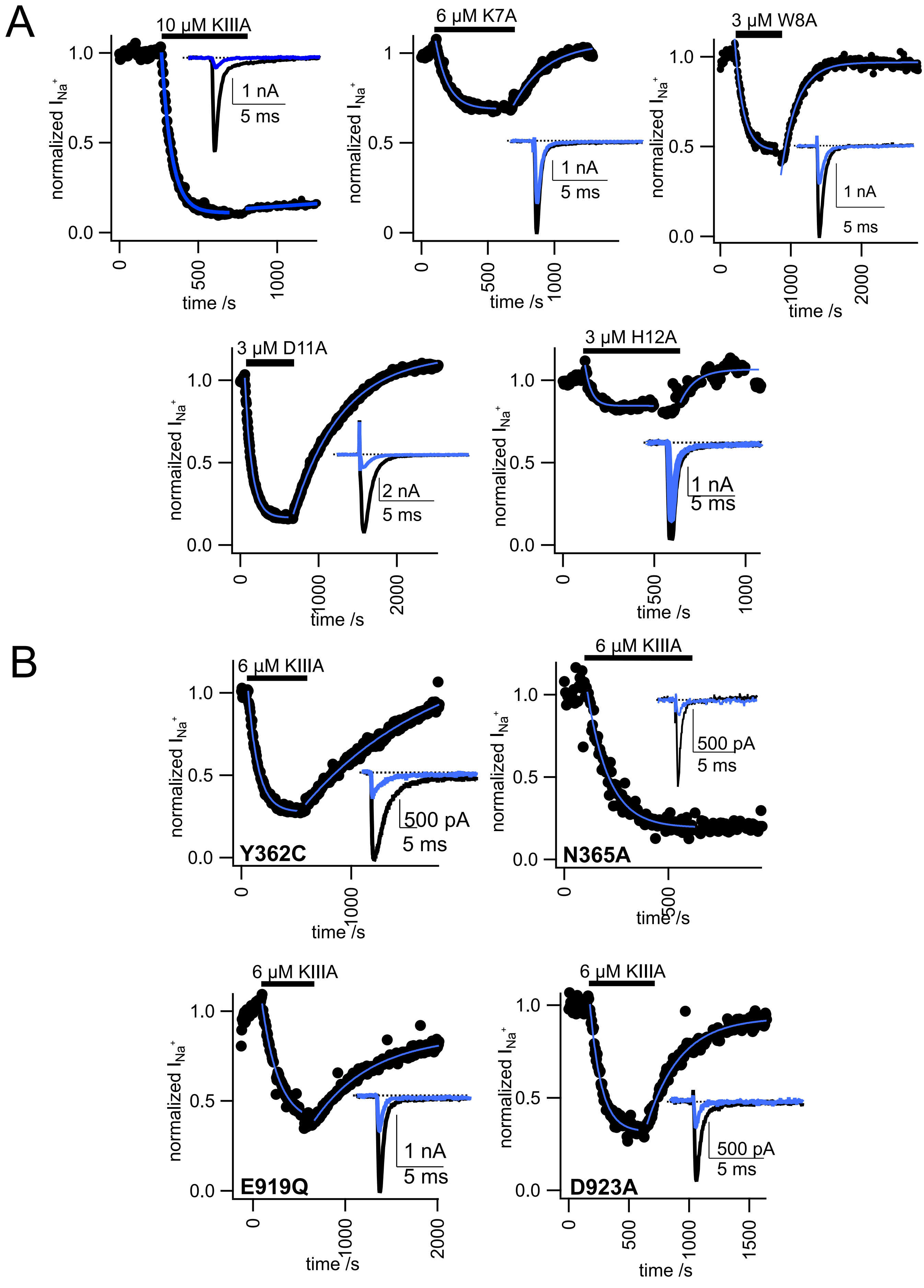

Supplementary Figure 3—Supplement 1

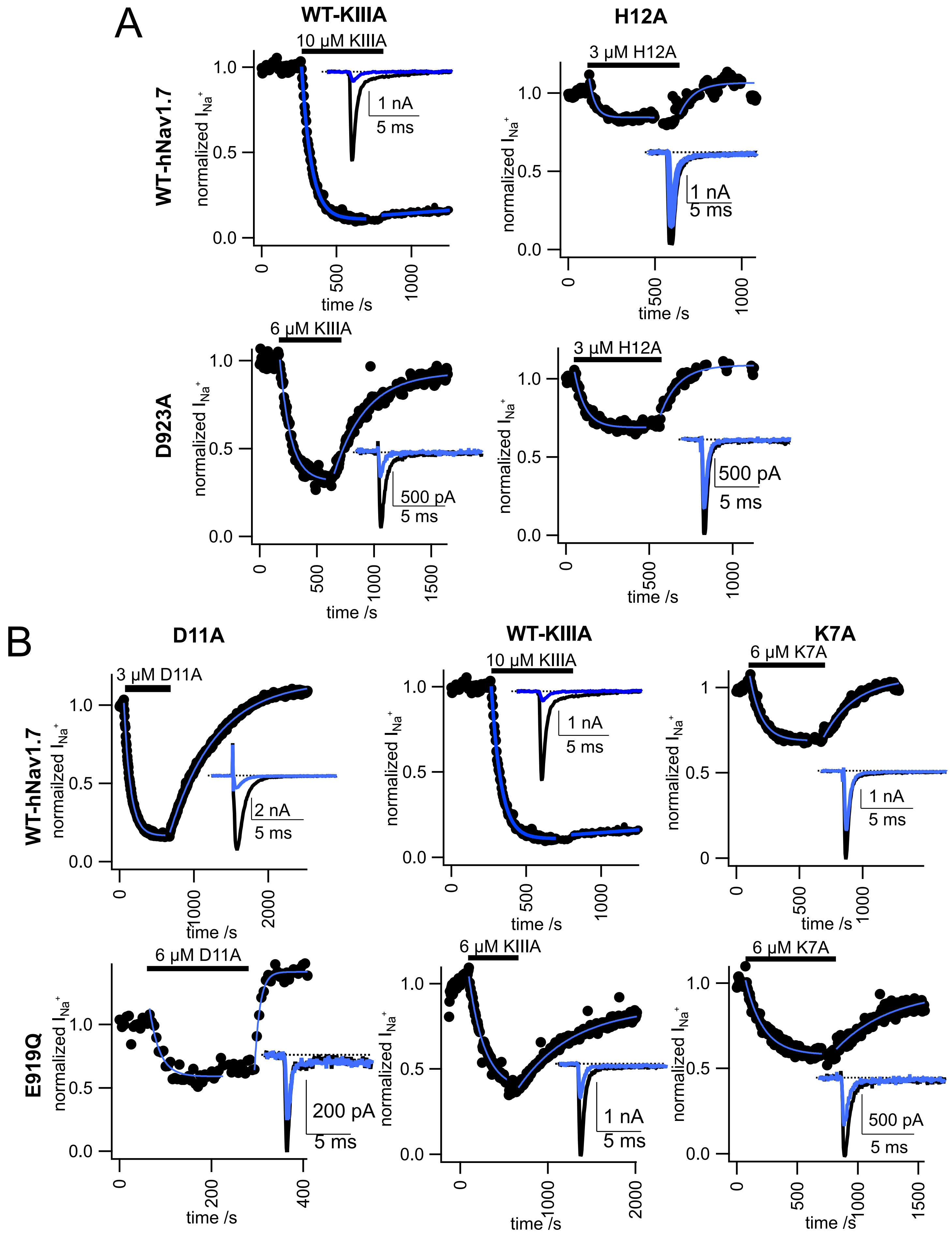

Supplementary Figure 4—Supplement 1

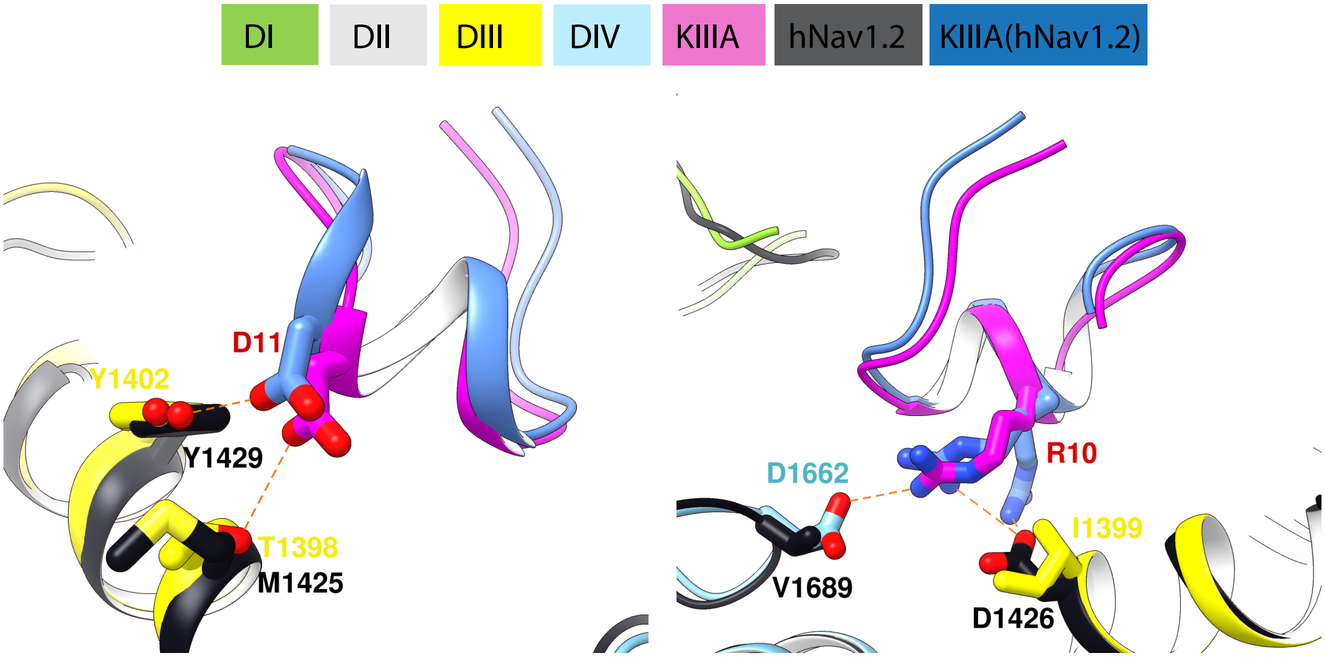

Supplementary Figure 5—Supplement 1

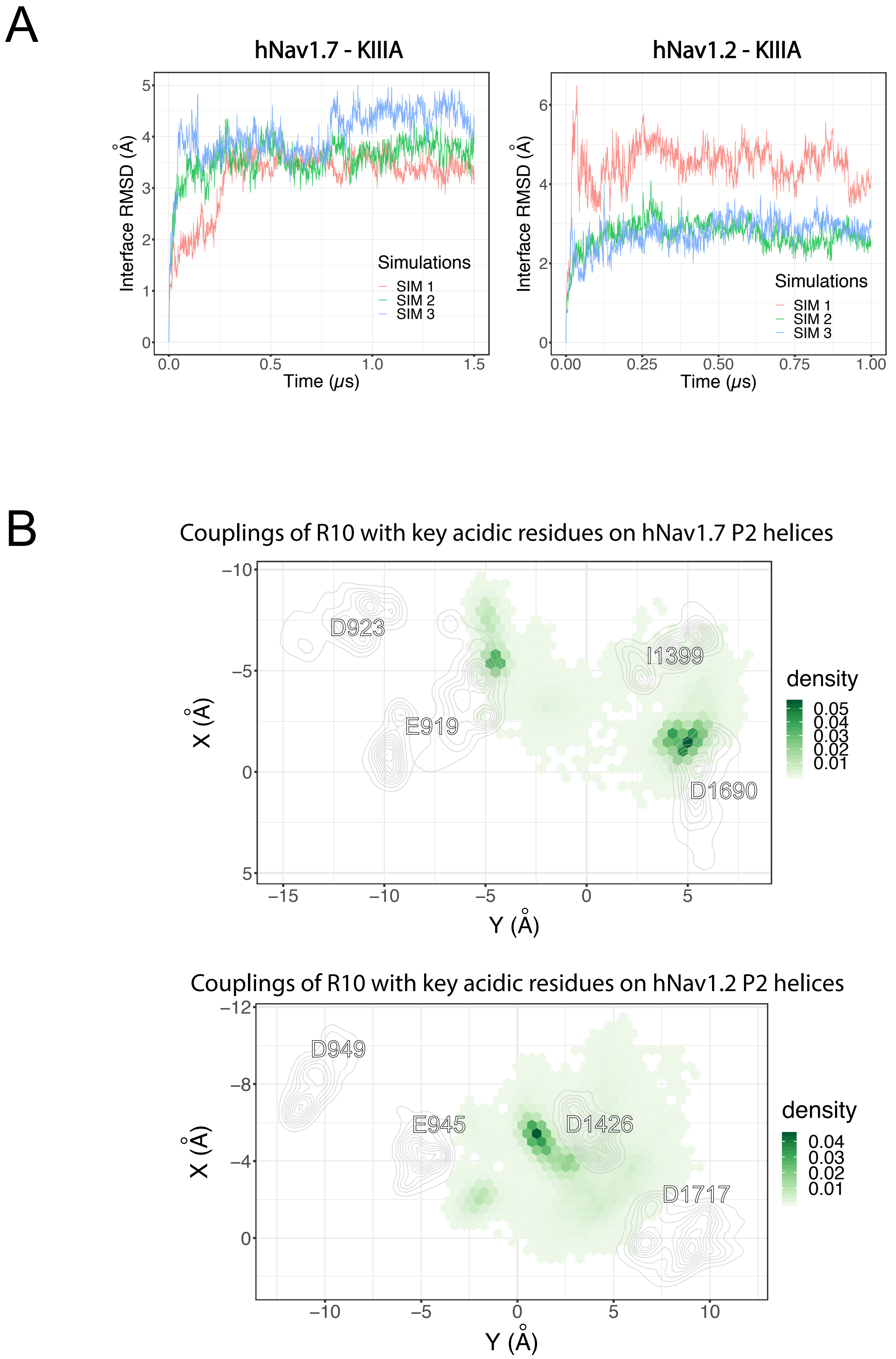

Supplementary Figure 5—Supplement 2

A

Representative atomic contacts of K7 and R10 with acidic residues on P2 helix of hNav1.7

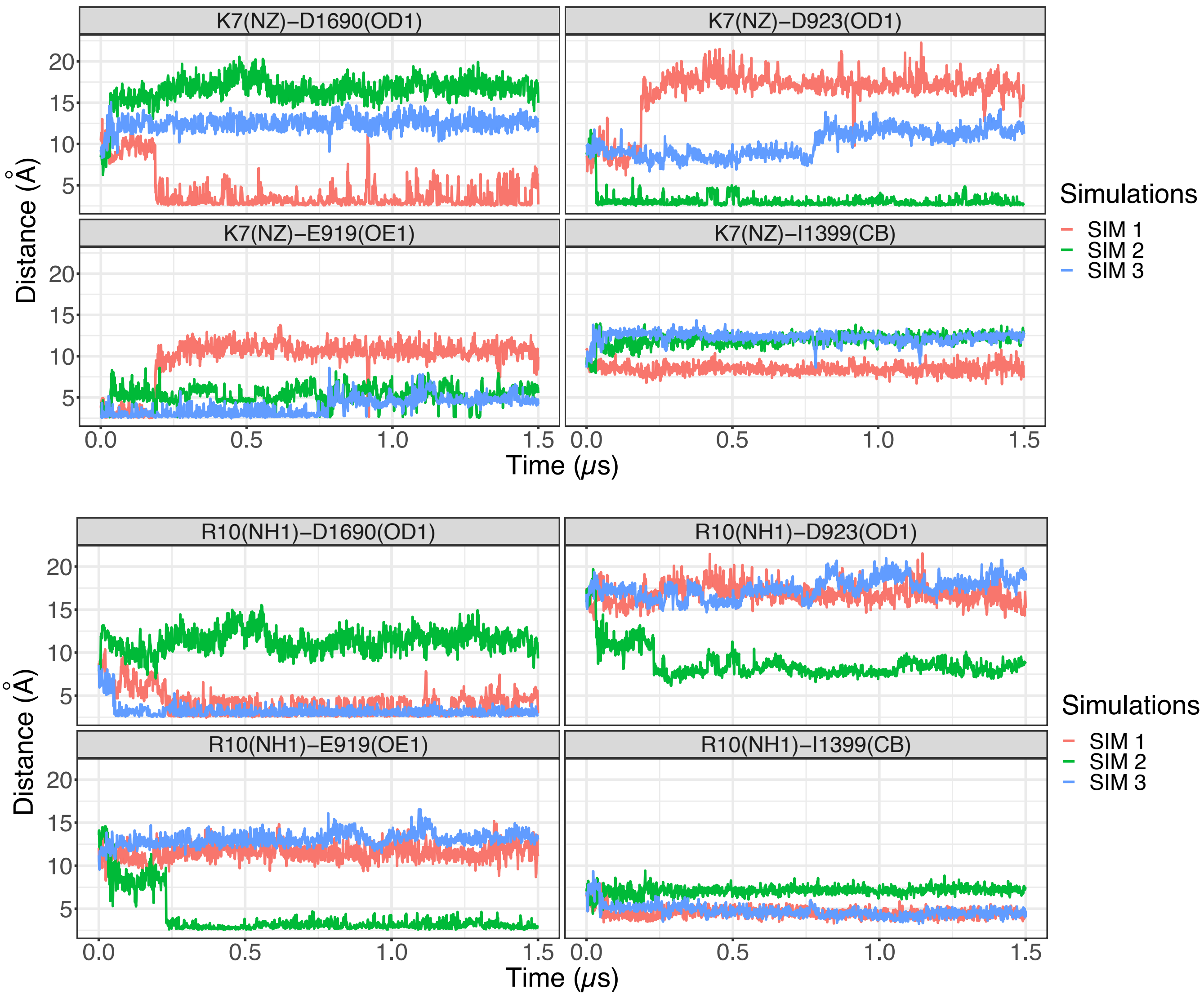

B

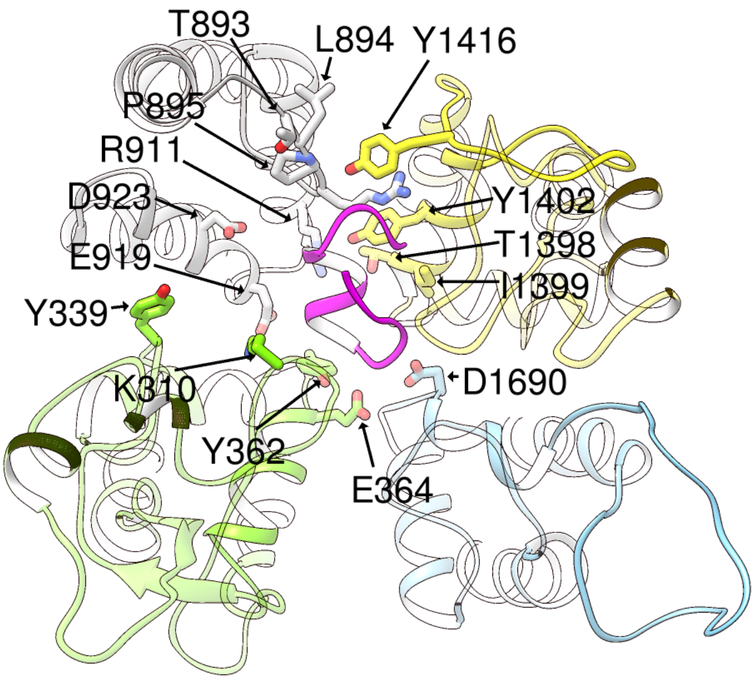

Supplementary Figure 5—Supplement 3

A

Representative atomic contacts of K7 and R10 with acidic residues on P2 helix of hNav1.2

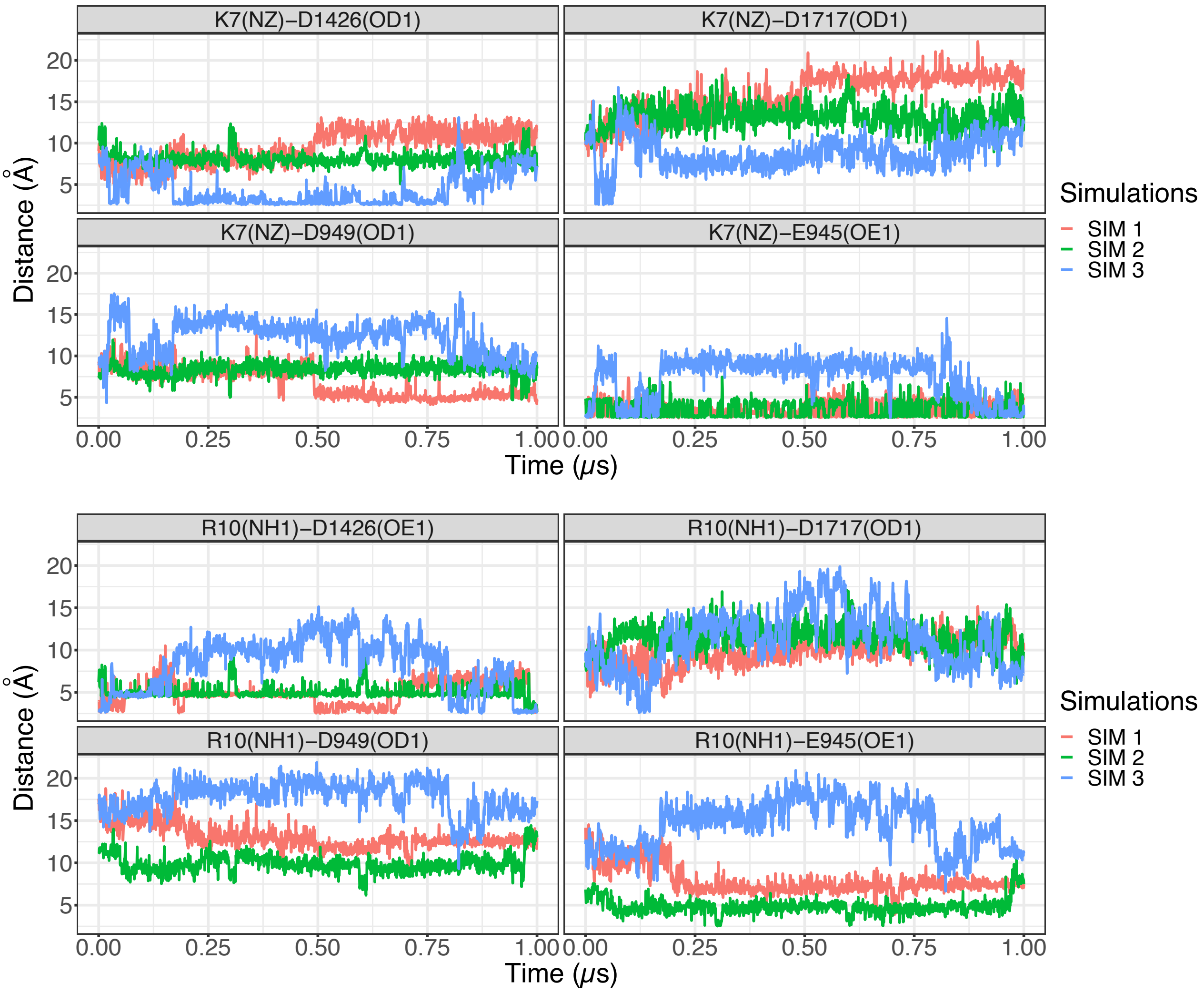

B

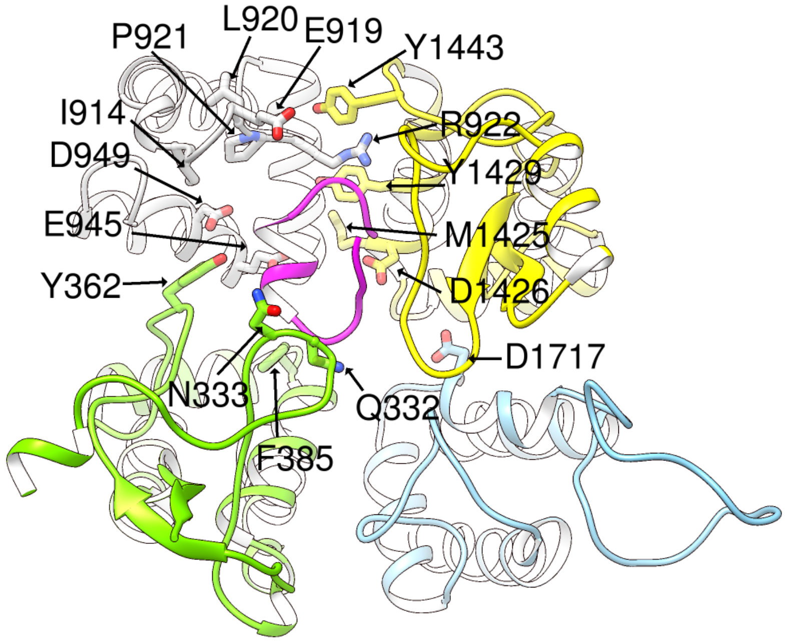

Supplement: Supplementary file 1 [file Presentation1.pdf]
